# Supplementary material for: Modelling genetic mosaicism of neurodevelopmental disorders in vivo by a Cre-amplifying fluorescent reporter
Source: Nat Commun. 2020 Dec 3;11:6194. doi: 10.1038/s41467-020-19864-w (PMC7713426; doi:10.1038/s41467-020-19864-w)
Supplement: Supplementary file 1 — Supplementary informations [file 41467_2020_19864_MOESM1_ESM.pdf]

Supplementary Information

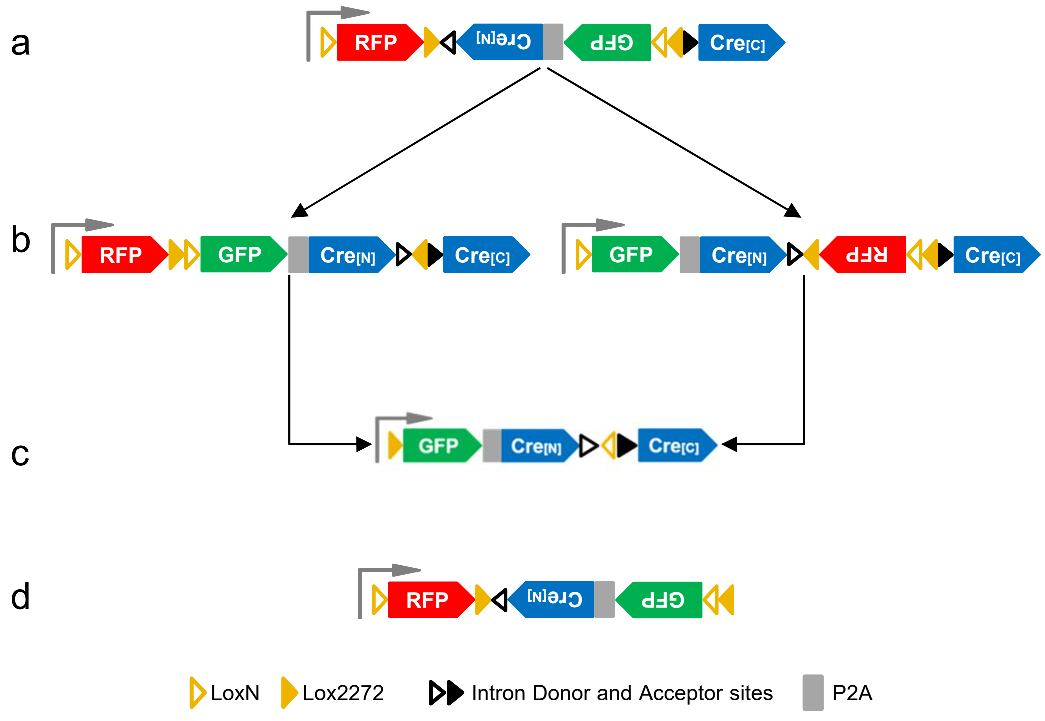

Supplementary Fig.1) Structure and recombination of Beatrix in the presence of Cre.

a, Schematic structure of Beatrix. Two steps of Cre-driven recombination are required to transform the RFP-expressing Beatrix, to the fully recombined GFP and Cre-expressing plasmid. **b**, The first step of recombination produces two alternative intermediate structures, depending on which lox pair is recombined first. The recombination of the Lox 2272 sites produces the intermediate shown on the left while the recombination of the Lox N sites leads to the structure shown on the right. **c**, The second recombination leads to the excision of RFP and the production of a structure that expresses only GFP and Cre. Notably, after the full recombination of Beatrix, only one Lox 2272 and one Lox N site are present, making any other recombination extremely unlikely. **d**, Structure of the control reporter: this plasmid includes the same lox sites as Beatrix, but the exon 2 of Cre gene has been removed. Therefore, it cannot lead to the amplification of Cre activity.

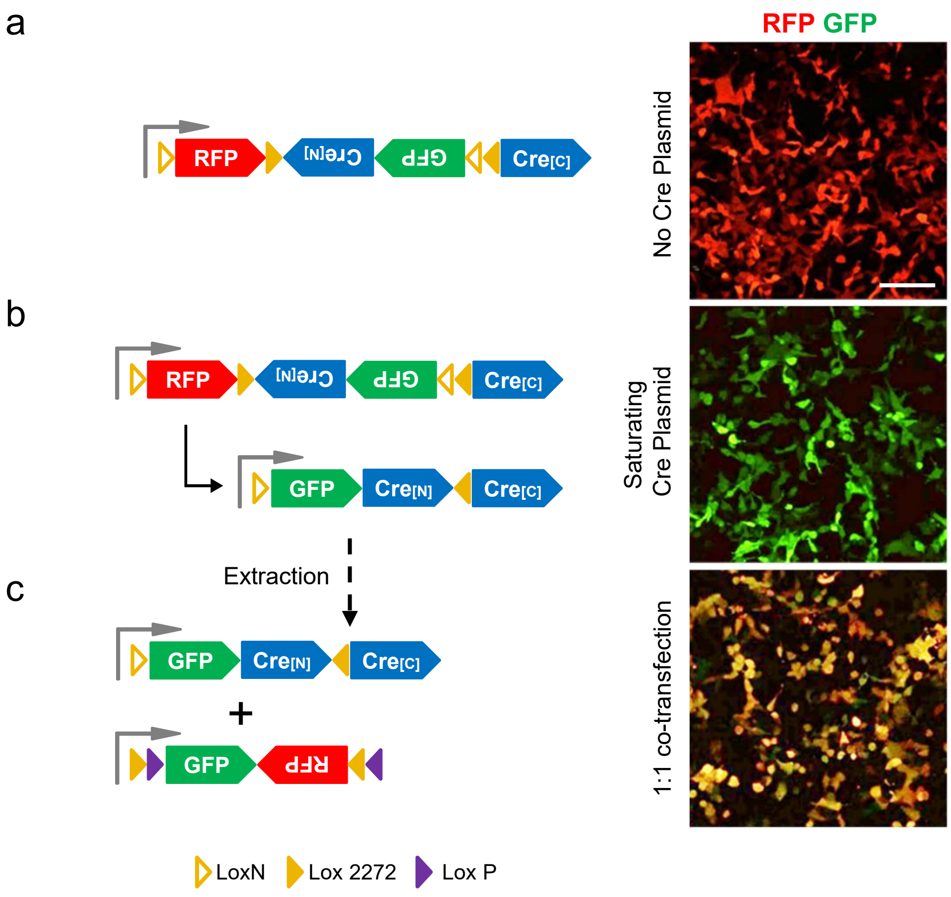

Supplementary Fig .2) The product of Beatrix recombination is a fully functional Cre recombinase.

a, The transfection of HEK 293T cells with Beatrix without the Cre controller produces a uniform population of cells expressing RFP only. **b**, Cells were co-transfected with Beatrix and a saturating concentration of Cre-expressing plasmid. This produced a full recombination of Beatrix thus leading to the expression of GFP only and of the reconstituted Cre. The DNA was extracted from this culture and we isolated and sequenced the fully recombined Beatrix thus confirming the expected structure depicted by the lower scheme. **c**, To prove the efficacy of Cre-activity supported by the recombined Beatrix, we used a simple Cre-switch reporter that expresses GFP in absence of Cre activity and, in presence of Cre, undergoes a two-step recombination involving Lox 2272 (yellow hollow triangles in the lower scheme) and Lox P sites (purple triangles) leading to the production of RFP. Purified Beatrix extracted from the culture shown in **b**, was co-transfected with the reporter in a 1:1 ratio. After transfection, all cells expresses both fluorescent proteins: the GFP provided by Beatrix and the RFP that is the reporter of the Cre-mediated recombination. Scale bar 50 µm.

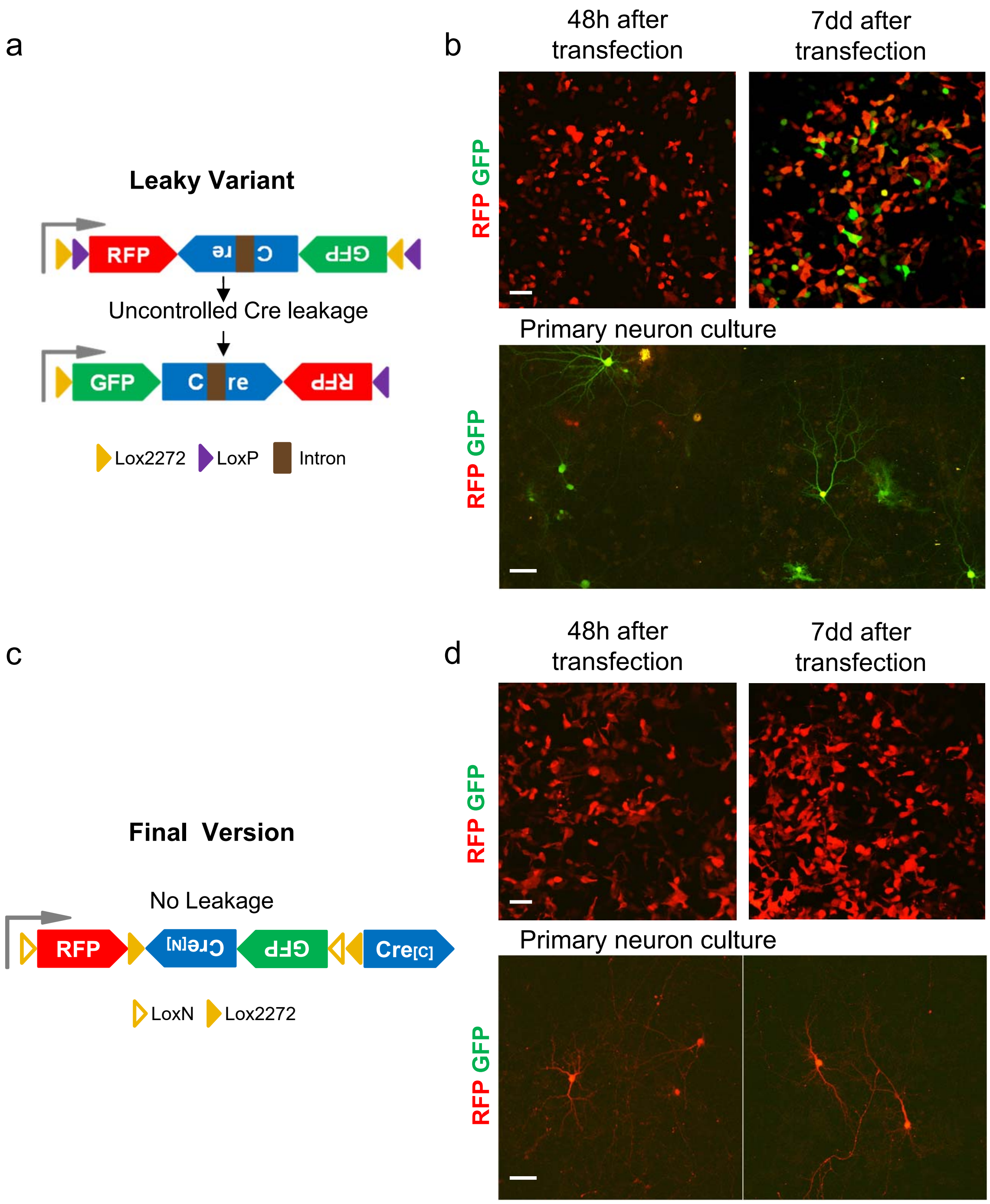

Supplementary Fig. 3) A previous variant of Beatrix showed a high non-induced self activation level.

a, An early version of the Beatrix construct was designed by inserting a GFP-P2A-Cre unit in antisense orientation using as a parent structure a Cre-Switch reporter (Addgene #37120). This plasmid should express an RFP before recombination and both GFP and Cre upon recombination. Unfortunately, this architecture suffers of an uncontrollable self-activation of the plasmid in absence of any Cre induction most likely due to antisense transcription of the Cre gene. Thus, a proper use of this construct is impossible since it is affected by uncontrollable leakage. **b**, 48 hours after transfection in HEK293T cells this construct resulted almost exclusively in RFP expressing cells (upper left panel) but, after 7 days from transfection, the leakage was very prominent and the amplification process was started in a large number of cells that switched from red to green (upper right panel). The leakage of this construct is more prominent in primary cultures of cortical neurons (bottom panel). **c**, The final Beatrix structure is characterized by the separation of the Cre gene in two exons that are orientated in opposite directions, preventing any unwanted transcription of the recombinase before induction. **d**, By adopting this strategy leakage dropped to zero and when transfected in both HEK293T and primary cortical neurons the sensor resulted exclusively in RFP expression. Scale bar 50 µm.

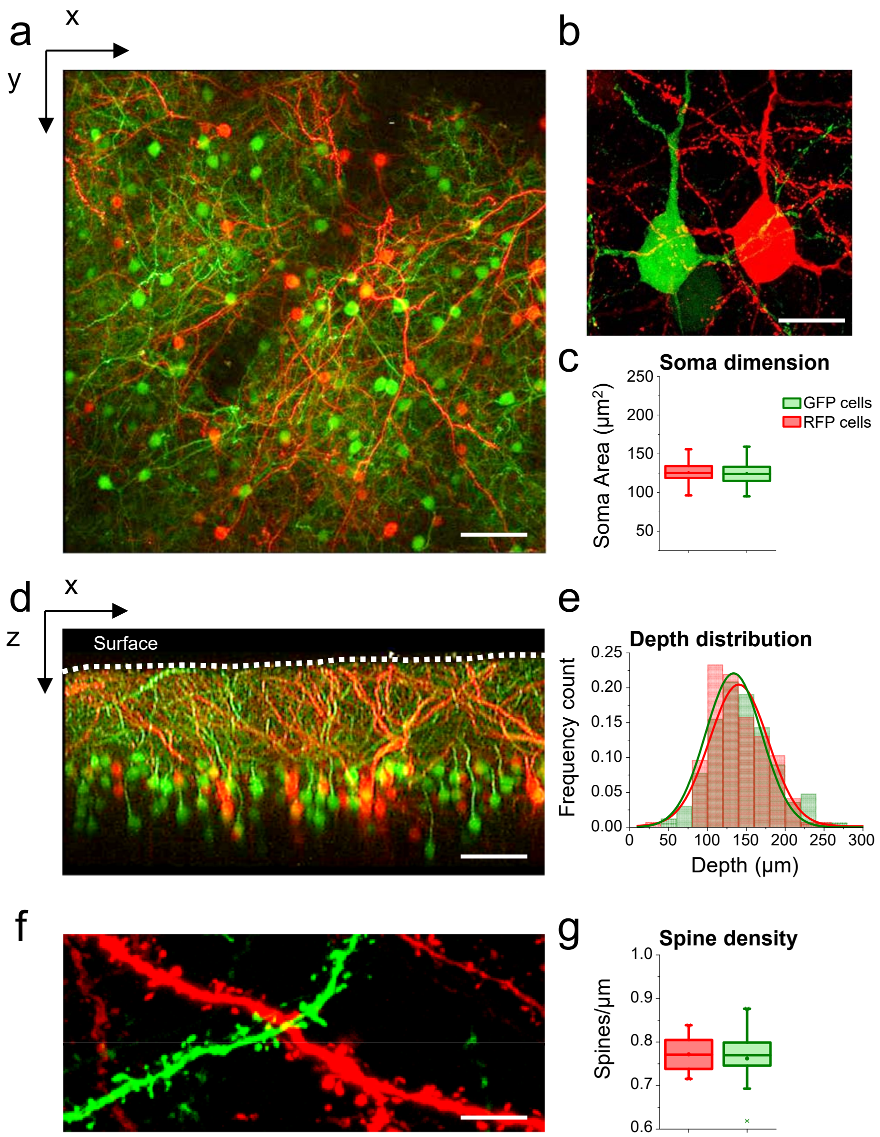

Supplementary Fig. 4) Cre amplification does not affect neuronal phenotype.

a, *In vivo* sparse mosaic at postnatal day 30 in cortical neurons on a wild type mouse obtained with a 1:50 Cre:Beatrix ratio. Scale bar 100 μm **b**, High magnification details of cell bodies from a WT mosaic. Scale bar 15 μm **c**, Soma dimension distribution of the two cellular subpopulations (RFP and GFP cells) in the WT-mosaic (n=3 animal; 254, 168 cells). No difference has been observed between RFP and GFP neurons in the average dimension of the cell bodies. **d**, Coronal projection of the *in vivo* Z-stack presented in a. Scale bar 100 μm. **e**, Distribution of the depth of the cortical neurons labeled with the mosaics in the WT animal. The two distributions show no significant difference in term of mean value and dispersion. (n=3 animal; 254, 168 cells). **f**, High magnification detail of dendritic structures from a WT-mosaic. Scale bar 10 μm **f**, The expression of Cre recombinase induced by Beatrix does not affect the spinogenesis. The plot shows how the two subpopulations have no significant difference in term of spine density in the granular layer. (n=3 animal; 254, 168 cells). All distributions resulted not significantly different at the 0.05 level (two-sided Kolmogorov-Smirnov test). **g**, Spine density quantification shows no significant difference between green and red cells dendritic compartments (two-sided Mann-Whitney U test).

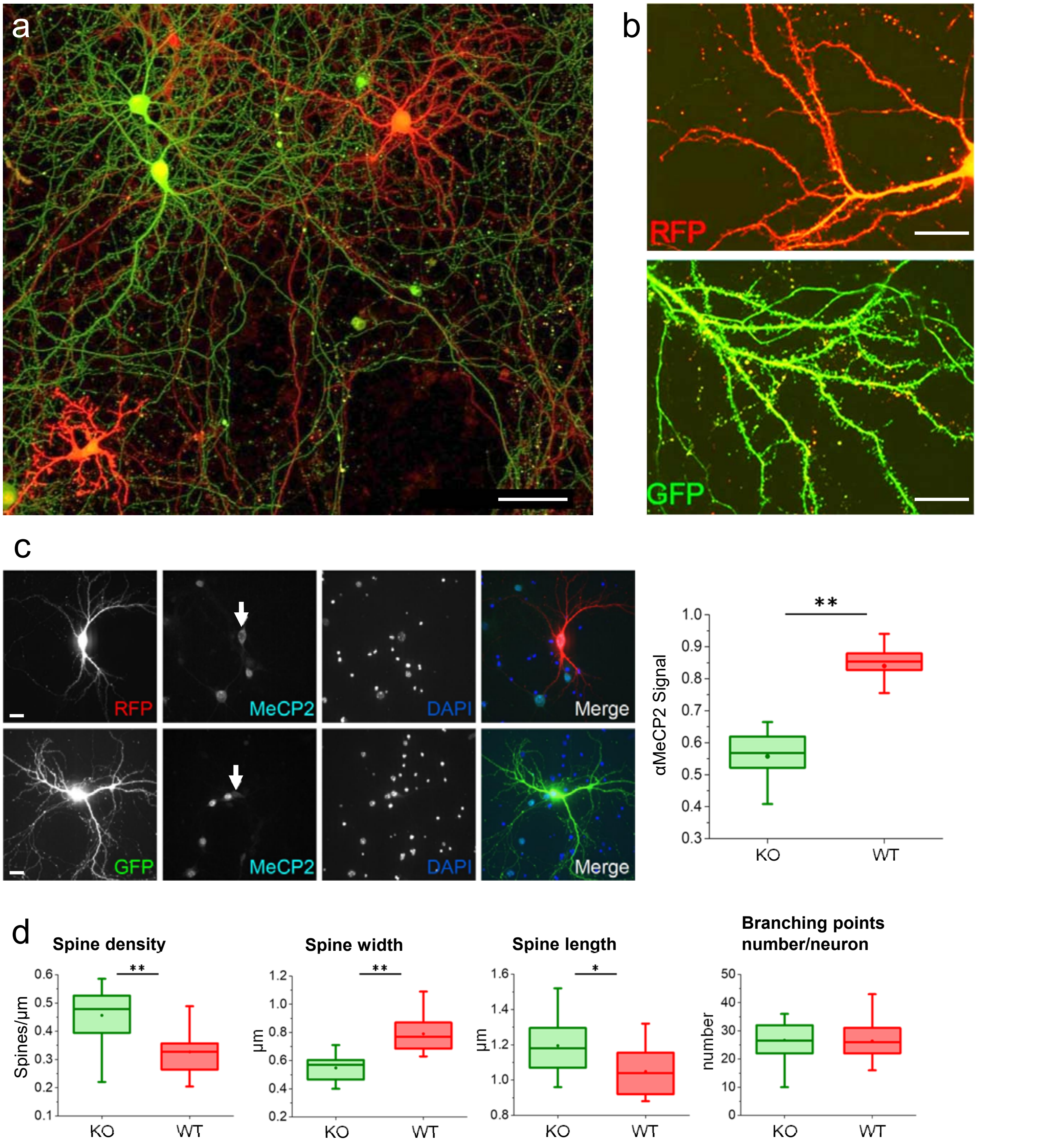

Supplementary Fig. 5) Cell-autonomous alterations of dendritic spines in a MeCP2 mosaics in neuronal cultures.

a, Sparse MeCP2 KO mosaics at 12DIV in dissociated mouse cortical neuron cultures obtained from $MeCP2^{flox}$ mice. Scale bar 50 μm **b**, High magnification details of dendrites from WT (top) and MeCP2-KO neurons (bottom). Scale bar 10 μm **c**, MeCP2 immunostaining quantification confirms the loss of MeCP2 in GFP^{+} cells, an its presence in RFP^{+} cells in the mosaics generated in cultured neurons (white arrows). These data confirms the possibility to extend the use of Beatrix-induced mosaics in a different experimental paradigm, including a different genomic target . Scale bar 20 μm. **d**, MeCP2 ablation in cultured cortical neurons leads to increased spine density with respect to the internal controls. This increase in spine number is characterized by morphological alterations, with spines presenting an average reduction of the width and a general increase in length. No difference has been observed between KO and WT neurons in the total number of branching points. (n neurons: 14 WT, 18 KO; two sided Mann-Whitney U test; * $p<0.05$, ** $p<0.01$).

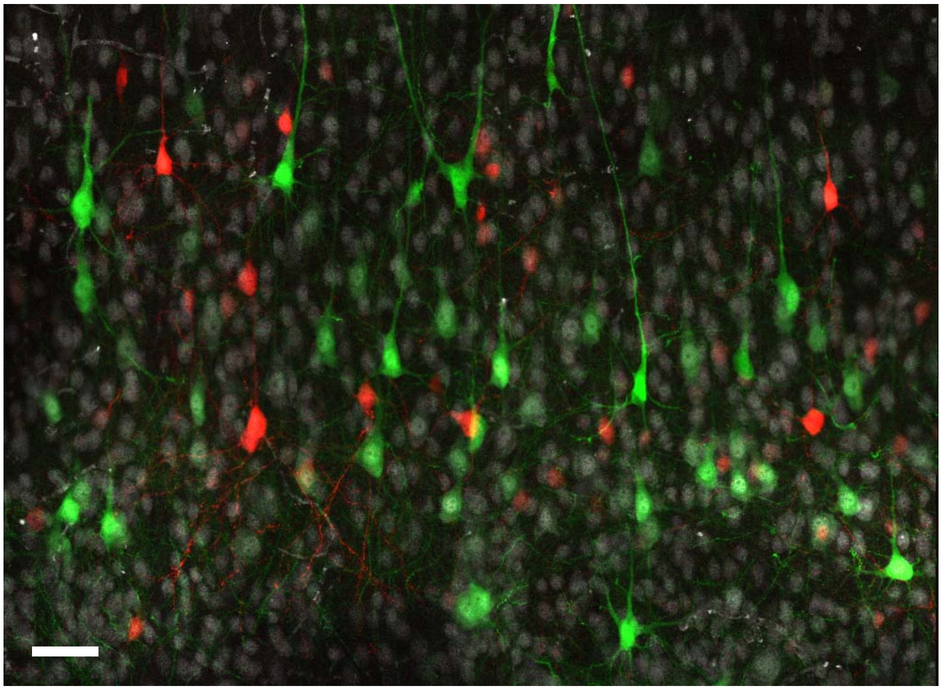

Supplementary Fig. 6) Immunofluorescence for the neuronal marker NeuN

Layers II/III acquired at the centre of the Beatrix fluorescent patch (red: WT pyramidal neurons; Green: PTEN-KO neurons). The section was processed for immunostaining against the neuronal marker NeuN (white). The cortical surface is on the image top, calibration bar 50 μm.

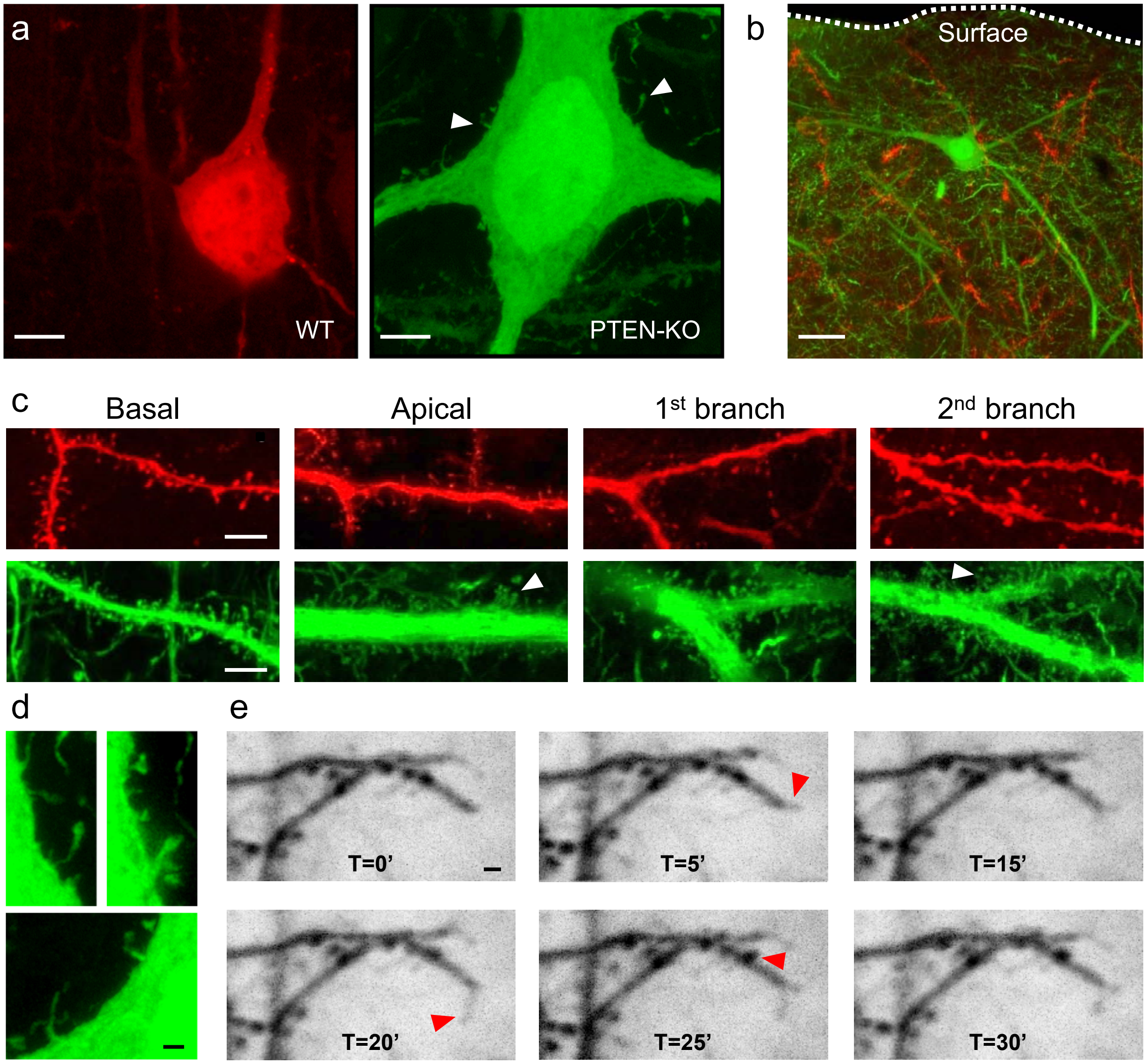

Supplementary Fig. 7) Details from the PTEN-KO mosaic model.

Two photon *in vivo* imaging of Beatrix-induced mosaics obtained by *in utero* electroporation and imaged at P30 in the somatosensory cortex. **a**, Cell bodies of a wild type and of a hypertrophic KO pyramidal neuron (Arrowheads point to abnormal somatic spines. Scale bar 5 μm). **b**, The alteration of the migratory phenotype induced by PTEN knockout in neuronal precursors occasionally results in a complete displacement of neurons. This image shows a cortical pyramidal neuron located in layer 1, approximately 40 μm from the brain surface, projecting its dendrites perpendicularly to the correct orientation (bar 20 μm). **c**, Representative images of dendritic compartments from wild type and PTEN knockout pyramidal neurons (lower panels). The images show how KO neurons exhibit the hypertrophic phenotype in all cellular compartments, along with a substantial increase in spine density and filopodia numbers (arrowheads; see also Fig. 4e quantification). Scalebar 5 μm **d**, High magnification images show the ectopic presence of dendritic spines and filopodia on the cell body surface (bar 1 μm). **e**, *In vivo* time lapse imaging of dendritic spines and of two dendrite terminals in layer 2 of the somatosensory cortex. These dendrites, shown in black for clarity, are PTEN-KO since they express GFP only. Imaging was performed at P25 and at this age it is expected that dendrites have reached a mature structure characterized by very limited short time spine motility and dendritic growth. These data show a tantalizing amount of plasticity, possibly associated to immature phenotype of the PTEN-KO dendrites also witnessed by the large number of filopodia as shown in panel c. Arrowheads point to structures of high motility.

Supplementary Table 1) Comparison of approaches for in vivo mosaicism modelling

Method	Beatrix	MASTR	iSuRe-Cre	Supernova	MADM	MASTR
**Reference**	This manuscript	Lao et al. Cell Reports 2012	Fernández-Chacón et al. Nature Comm. 2019	Lin et al. Nature Methods 2018	Zong et al. Cell 2005	Kim et al. Cell 2019
**Sensitivity as Cre Reporter**	Extremely higher than conventional reporters due to amplification	Not a Cre reporter	Lower than standard ROSA26 reporters	Standard AAV levels (not devised to be used as a Cre reporter)	Standard ROSA26 reporter levels	Standard ROSA26 reporter levels (needs to be coupled with a reporter mouse strain)
**Reliability as a floxed genes status Reporter**	**VERY HIGH:** False positives and false negatives are virtually absent	**HIGH:** Low false positives, Standard false negative levels	**VARIABLE:** No False positives, presence of false negatives	Not tested with floxed strains	**VARIABLE:** False positives and false negatives depends on Cre promoter leakage	**NOT** devised to be used as a WT/KO mosaic tool
**Simplicity of the system**	**HIGH** (Cre only)	**LOW** (Cre+Flp)	**HIGH** (Cre only)	**VERY LOW** (Cre+tetON+ Flp/ Dre)	**HIGH** (Cre only)	**LOW** (Cre+Flp)
**Tunable Mosaic generation**	**TUNABLE WT/KO** mosaics and sparse labelling in floxed or WT strains/lines	Exclusively with floxed mice strains expressing Flp-ERT2 through low tamoxifen injections	Proposed but not addressed	Tunable sparse labelling *in vivo*	**NOT TUNABLE** Relies on Cre-guided inter-chromosomal mitotic recombination	Tunable WT/GOF mosaics and sparse labelling only on reporter mice strains through RMCE
**Unwanted/ Leaky activity**	**ABSENT**	**LEAKY** in the mouse male germline	**VERY LEAKY** in transfected cells. Still present in the mouse strain	**VERY LEAKY:** The system relies on the leaky activity of the Tet-TRE system	**VARIABLE:** depends on the leakage of the promoter driving Cre expression	**LIMITED:** Prolonged recombinase expression may lead to in/out hopping of the donor cargo
