# Supplementary material for: Modelling genetic mosaicism of neurodevelopmental disorders in vivo by a Cre-amplifying fluorescent reporter
Source: Nat Commun. 2020 Dec 3;11:6194. doi: 10.1038/s41467-020-19864-w (PMC7713426; doi:10.1038/s41467-020-19864-w)
Supplement: Supplementary file 2 — Reporting Summary [file 41467_2020_19864_MOESM2_ESM.pdf]

nature research

Corresponding author(s): Gian Michele Ratto
Francesco Trovato

Last updated by author(s): Sep 30, 2020

Reporting Summary

Nature Research wishes to improve the reproducibility of the work that we publish. This form provides structure for consistency and transparency in reporting. For further information on Nature Research policies, see our Editorial Policies and the Editorial Policy Checklist.

Statistics

For all statistical analyses, confirm that the following items are present in the figure legend, table legend, main text, or Methods section.

n/a	Confirmed	
☐	☒	The exact sample size (n) for each experimental group/condition, given as a discrete number and unit of measurement
☐	☒	A statement on whether measurements were taken from distinct samples or whether the same sample was measured repeatedly
☐	☒	The statistical test(s) used AND whether they are one- or two-sided<br>*Only common tests should be described solely by name; describe more complex techniques in the Methods section.*
☐	☒	A description of all covariates tested
☐	☒	A description of any assumptions or corrections, such as tests of normality and adjustment for multiple comparisons
☐	☒	A full description of the statistical parameters including central tendency (e.g. means) or other basic estimates (e.g. regression coefficient) AND variation (e.g. standard deviation) or associated estimates of uncertainty (e.g. confidence intervals)
☐	☒	For null hypothesis testing, the test statistic (e.g. F, t, r) with confidence intervals, effect sizes, degrees of freedom and P value noted<br>*Give P values as exact values whenever suitable.*
☐	☒	For Bayesian analysis, information on the choice of priors and Markov chain Monte Carlo settings
☐	☒	For hierarchical and complex designs, identification of the appropriate level for tests and full reporting of outcomes
☐	☒	Estimates of effect sizes (e.g. Cohen's d, Pearson's r), indicating how they were calculated

Our web collection on statistics for biologists contains articles on many of the points above.

Software and code

Policy information about availability of computer code

Data collection	Images were analyzed with the software Fiji, a distribution of image analysis software ImageJ version 1.52t.<br>Electrophysiology data were recorded using a custom software written with LabView 13.2.
Data analysis	Data analysis was performed with Origin 9.0 for what concerns data collected from imaging.<br>lectrophysiology data were analyzed using a custom app written with Matlab 2020a. Plots were produced with either Matlab 2020a (spectrograms) or Origin 9.0.

For manuscripts utilizing custom algorithms or software that are central to the research but not yet described in published literature, software must be made available to editors and reviewers. We strongly encourage code deposition in a community repository (e.g. GitHub). See the Nature Research guidelines for submitting code & software for further information.

Data

Policy information about availability of data

All manuscripts must include a data availability statement. This statement should provide the following information, where applicable:

- Accession codes, unique identifiers, or web links for publicly available datasets
- A list of figures that have associated raw data
- A description of any restrictions on data availability

The raw spreadsheet on which the figures are based are available as supplementary material. The complete datasets generated during and/or analyzed during the current study are available from the corresponding author on reasonable request.

Field-specific reporting

Please select the one below that is the best fit for your research. If you are not sure, read the appropriate sections before making your selection.

- [x] Life sciences
- [ ] Behavioural & social sciences
- [ ] Ecological, evolutionary & environmental sciences

For a reference copy of the document with all sections, see nature.com/documents/nr-reporting-summary-flat.pdf

Life sciences study design

All studies must disclose on these points even when the disclosure is negative.

Sample size	Sample size was chosen based on preliminary experiments that showed what degree of variability was present in the samples.<br>For in vivo experiments, sample sizes were chosen to represent at least 3 different animals, with more than 3 sections each and more than 3 fields of acquisition per section, with a 300-500 cells per animal.<br>For cell culture experiments, 3-4 replicates were performed per condition and each replicate would be composed of 500-1000 cells.<br>The number of animals or cells has been reported in each figure legend (n).
Data exclusions	No collected data were excluded from the analysis.
Replication	All attempts at replicating the results described in the paper were successful, with the exception of failed in utero electroporations or failed cell transfections. In these cases, no fluorescent cells were present, therefore no data were collected from those plates or animals.
Randomization	For electroporation experiments, female mice to be electroporated were chosen randomly in an age range of 2-6 months, they were mated with randomly chosen male mice and then, if pregnant, electroporated at E15. All born pups were then analyzed, except for cases of complete failure of electroporation on the pup (i.e. lack of fluorescent cells).<br>For cell experiments, in each session all groups of plasmids were transfected in cells belonging to the same plates, which were split into two groups on the day of transfection.
Blinding	In animal experiments on PTEN KO mice, no blinding was used because PTEN knockout mice phenotype and wild type mice phenotype ere clearly distinguishable.<br>In experiments on cell lines and electroporations of Beatrix plamid on wild type mice, blinding was not used because Beatrix and control plasmid-treated cells were clearly disinguishable, as clearly observable in figures 1 and 2.

Reporting for specific materials, systems and methods

We require information from authors about some types of materials, experimental systems and methods used in many studies. Here, indicate whether each material, system or method listed is relevant to your study. If you are not sure if a list item applies to your research, read the appropriate section before selecting a response.

Materials & experimental systems

n/a	Involved in the study	
	x	Antibodies
	x	Eukaryotic cell lines
x		Palaeontology and archaeology
	x	Animals and other organisms
x		Human research participants
x		Clinical data
x		Dual use research of concern

Methods

n/a	Involved in the study	
x		ChIP-seq
x		Flow cytometry
x		MRI-based neuroimaging

Antibodies

Antibodies used	Primary antibodies:<br>αMeCP2 (Cell Signaling, #3465), αCre (Sigma-Aldrich, C7988), αNeuN (Sigma-Aldrich, MAB377) and αPTEN (Cell Signaling, 9559)<br>Secondary antibodies:<br>Fluorophore-conjugated (Jackson ImmunoResearch Laboratories) secondary antibodies were used for immunocytochemistry; Alexa Fluor-conjugated secondary antibodies (Abcam AF405, AF647) were used for immunohistochemistry.
Validation	Validation was provided by the vendor for all antibodies used in this study.

Eukaryotic cell lines

Policy information about cell lines

Cell line source(s)	HEK-293 cells were a kind gift from Francesco Gobbo, Scuola Normale Superiore (Pisa, Italy).<br>NIH3T3 cells were a kind gift from Melissa Santi, CNR Nanoscience Institute (Pisa, Italy)
Authentication	None of the cell lines used were authenticated by the authors.
Mycoplasma contamination	No mycoplasma contamination was found by PCR analysis.
Commonly misidentified lines<br>(See ICLAC register)	No commonly misidentified cell line was used.

Animals and other organisms

Policy information about studies involving animals; ARRIVE guidelines recommended for reporting animal research

Laboratory animals	Mice have been housed in animal facilities adopting a 12 light/12 dark cycle, with temperatures of 18-23 C and 40-60% humidity, and have been fed ad libitum.<br>Used strains:<br>-Inbred C57BL/6J mice (JAX stock #000664) were obtained from the Jackson Laboratory (Bar Harbor, ME, USA).<br>-PTENflox mice (JAX stock #004597) were obtained from the Jackson Laboratory (Bar Harbor, ME, USA).<br>-Outbred CD-1 IGS adult pregnant female mice (strain code 022) were obtained from Charles River Laboratories (Wilmington, MA, USA).<br>-Mecp2lox mice72 maintained on a C57BL6/J background (MMRRC; B6; 129S4- Mecp2tm1Jae/Mmucd; stock number: 011918-UCD).<br>All mice used for experiments were randomly chosen, without distinction for sex (apart from in utero electroporation, which is performed on female mice). Pregnant mice for in utero electroporation were 2-6 months old and embryos were electroporated at gestation day E15.5; electroporated pups were imaged at age P28-32.
Wild animals	The study did not involve wild animals.
Field-collected samples	The study did not involve field-collected animals.
Ethics oversight	Protocols for animal experiments were approved by the Italian Superior Institute of Health (Rome, Italy) and by a committee for animal care evaluation (OPBA, CNR Research Institute, Pisa).

Note that full information on the approval of the study protocol must also be provided in the manuscript.
